# The Virulence of *Escherichia coli* O157:H7 Isolates in Mice Depends on Shiga Toxin Type 2a (Stx2a)-Induction and High Levels of Stx2a in Stool

**DOI:** 10.3389/fcimb.2020.00062

**Published:** 2020-02-26

**Authors:** Jocelyn R. Hauser, Rama R. Atitkar, Courtney D. Petro, Rebecca L. Lindsey, Nancy Strockbine, Alison D. O'Brien, Angela R. Melton-Celsa

**Affiliations:** ^1^Department of Microbiology and Immunology, Uniformed Services University of the Health Sciences, Bethesda, MD, United States; ^2^Centers for Disease Control and Prevention, Atlanta, GA, United States

**Keywords:** *Escherichia coli*, O157:H7, Shiga toxin, Stx2, phage, *recA*, mouse model

## Abstract

In this study we compared nine Shiga toxin (Stx)-producing *Escherichia coli* O157:H7 patient isolates for Stx levels, *stx*-phage insertion site(s), and pathogenicity in a streptomycin (Str)-treated mouse model. The strains encoded *stx*_2a_, *stx*_1a_ and *stx*_2a_, or *stx*_2a_ and *stx*_2c_. All of the strains elaborated 10^5^-10^6^ cytotoxic doses 50% (CD_50_) into the supernatant after growth *in vitro* as measured on Vero cells, and showed variable levels of increased toxin production after growth with sub-inhibitory levels of ciprofloxacin (Cip). The *stx*_2a_+*stx*_2c_+ isolates were 90–100% lethal for Str-treated BALB/c mice, though one isolate, JH2013, had a delayed time-to-death. The *stx*_2a_+ isolate was avirulent. Both an *stx*_2a_ and a *recA* deletion mutant of one of the *stx*_2a_+*stx*_2c_+ strains, JH2010, exhibited at least a three-log decrease in cytotoxicity *in vitro* and both were avirulent in the mice. Stool from Str-treated mice infected with the highly virulent isolates were 10- to 100-fold more cytotoxic than feces from mice infected with the clinical isolate, JH2012, that made only Stx2a. Taken together these findings demonstrate that the *stx*_2a_-phage from JH2010 induces to higher levels *in vivo* than does the phage from JH2012. The *stx*_1a_+*stx*_2a_+ clinical isolates were avirulent and neutralization of Stx1 in stool from mice infected with those strains indicated that the toxin produced *in vivo* was primarily Stx1a. Treatment of mice infected with Stx1a+Stx2a+ isolates with Cip resulted in an increase in Stx2a production *in vivo* and lethality in the mice. Our data suggest that high levels of Stx2a in stool are predictive of virulence in mice.

## Introduction

Shiga toxin (Stx)-producing *E. coli* (STEC) O157:H7 is a foodborne pathogen estimated to cause more than 265,000 episodes of diarrheal illnesses each year in the United States, with more than 3,600 hospitalizations and 30 deaths (Scallan et al., 2011). The estimated infectious dose for O157:H7 is from 10 to 700 organisms (Tuttle et al., 1999; Hara-Kudo and Takatori, 2011). Spontaneous resolution of the infection usually occurs in about 85% of patients. However, 3–20% of people with confirmed *E. coli* O157 infection develop hemolytic uremic syndrome (HUS), a sequela characterized by acute kidney failure, thrombocytopenia, and hemolytic anemia (Tarr et al., 2005). Children are disproportionately affected as over 90% of STEC-associated HUS cases occur among children under the age of 5 (Friedrich et al., 2002; Spinale et al., 2013). In addition, the use of antibiotic therapy in patients infected with STEC is linked to an increased risk for the HUS (Freedman et al., 2016). The long-term sequelae of STEC-related HUS in both children and adults can include renal, neurological, pulmonary and cardiac complications (Tarr et al., 2005). The only recommended treatment for STEC-related HUS involves supportive therapy, such as intravenous volume expansion, which has been shown to improve long-term renal outcome (Ake et al., 2005).

The symptoms and sequelae of STEC infection are the result of tissue damage caused by Stx. Stx is a potent AB5 toxin that binds to cells that express the toxin receptor, globotriaosylceramide (GB3) and inhibits protein synthesis in the target cell (see review Melton-Celsa, 2014). There are two antigenically distinct prototype Stxs: Stx1a and Stx2a. There are also subtype variations within each toxin type. Stx subtypes associated with human clinical disease include Stx1a, Stx2a, Stx2c and Stx2d. The Stx operon is encoded on a lysogenic lamdoid-like bacteriophage integrated within the bacterial genome. Phage and host cell factors influence toxin production and release. Although there is a basal level of Stx produced, likely due to spontaneous induction of the *stx*-phage(s) within a strain, induction of the *stx*-phage is linked to the bacterium's stress response. When the bacterial cell is grown in the presence of the antibiotic ciprofloxacin (Cip), it undergoes the stress response due to DNA damage and the phage lytic cycle is induced. The latter process is likely the explanation for the increased risk for the development of the HUS after treatment of STEC patients with antibiotics (Freedman et al., 2016).

Although there is a new study that links several single nucleotide polymorphisms in the human genome with an increased susceptibility to post-STEC HUS (Kallianpur et al., 2018), no well-defined patient-specific factor(s) has been found that can be used to predict whether a patient will develop O157:H7-associated post-diarrheal HUS. However, the risk of developing HUS is increased for children under 10 years age and in patients of any age who develop leukocytosis, experience treatment delays, or receive antibiotics and/or anti-motility agents (Tarr et al., 2005). Although the presence of the locus of enterocyte effacement (LEE) in an STEC isolate, the production of specific Stx subtypes, and *E. coli* O157:H7 clade classification have been associated with STEC that cause severe human disease (Manning et al., 2008; Neupane et al., 2011; Amigo et al., 2015; Bunger et al., 2015), no other pathogen-specific factors have been identified that can be used to predict the severity of STEC related disease. The focus of this study was to determine whether factors, such as *stx*-subtype, Stx protein levels, *stx*-phage insertion sites, and virulence of O157:H7 clinical isolates in a mouse model correlated with disease presentation in patients. Although we were unable to identify specific bacterial factors that corresponded to patient clinical outcome data, we did find that that pathogenesis in a streptomycin (Str)-treated mouse model correlated with elevated induction of the *stx*_2a_-phage *in vivo*.

## Results

### Stx Subtypes, *stx*-Phage Insertion Site, and Virulence Gene Profiles of Clinical O157:H7 Strains Do Not Correlate With Clinical Presentation

The Stx subtypes, *stx*-phage insertion sites, and virulence genes profiles of nine clinical O157:H7 isolates from patients that had non-bloody diarrhea (NBD, *n* = 3), bloody diarrhea (BD, *n* = 3), or bloody diarrhea that progressed to HUS (*n* = 3) are summarized in Table 1. The toxin genotypes of the O157:H7 isolates were *stx*_1a_ and *stx*_2a_ (*n* = 3), *stx*_2a_ (*n* = 1), or *stx*_2a_ and *stx*_2c_ (*n* = 5). Our finding that five of the strains were *stx*_2a_+*stx*_2c_+ was not surprising to us because many O157:H7 isolates in the USA and Finland have that genotype (Eklund et al., 2002; Tarr et al., 2019), including the strain from the 2006 spinach outbreak in the USA (Uhlich et al., 2008). Of the nine known loci in which *stx* phages insert into the host bacterial chromosome (Bonanno et al., 2015), we observed that for strains lysogenized by both *stx*_1a_- and *stx*_2a_-phages, the *stx*_1a_ phage occupied *yehV*, and the *stx*_2_-phage occupied *wrbA*. In the *stx*_2a_*stx*_2c_ isolates, the *stx*_2c_-phage was in *sbcB* and the *stx*_2a_-phage was in a*rgW*. The finding that the *stx*_2a_ phage was in *argW* rather than in *wrbA* has been shown for 40–77% of human O157:H7 isolates (Shaikh and Tarr, 2003; Shringi et al., 2012). Only one strain, JH2012, was lysogenized by a single *stx*-phage, and that *stx*_2a_-phage was also inserted in *argW*. Although the *stx*_2a_+*stx*_2c_+ strains did not have *stx*_1_, *yehV* was occupied by an *stx*_1_-like defective phage, a finding that has been described for some other STEC (Shaikh and Tarr, 2003).

**Table 1:** Summary of clinical outcomes, *stx*-subtyping, clades, and phage insertion sites for nine O157:H7 clinical isolates.

**Strain**	**CDC ID^a^**	**Clinical outcome**	**Hospitalized?**	**Age**	***stx*-subtype(s)**	**Clade**	***stx*-phage insertion sites**			
							***stx*_1_**	***stx*_2_**		
							***yehV^b^***	***wrbA***	***sbcB***	***argW***
JH2014	2009C-3554	non-BD	N	0–4	*stx*_1a_*stx*_2a_	2	+	+	–	–
JH2015	2009C-4207	HUS	Y	5–9	*stx*_1a_*stx*_2a_	2	+	+	–	–
JH2018	2010C-3347	non-BD	N	20–29	*stx*_1a_*stx*_2a_	ND^c^	+	+	–	–
JH2010	06-3462	non-BD	Y	0–4	*stx*_2a_*stx*_2c_	8	–	–	+	+
JH2011	08-3914	HUS (outbreak)	Y	0–4	*stx*_2a_*stx*_2c_	8	–	–	+	+
JH2016	2009C-4687	HUS	Y	0–4	*stx*_2a_*stx*_2c_	8	–	–	+	+
JH2017	2010C-3142	BD (outbreak)	N	50–59	*stx*_2a_*stx*_2c_	8	–	–	+	+
JH2013	2009C-3378	BD	N	5–9	*stx*_2a_*stx*_2c_	8	–	–	+	+
JH2012	08-3918	BD	N	40–49	*stx*_2a_	8	–	–	–	+

*+stx-phage insertion*.

*–No stx-phage insertion*.

a *ID number assigned by the CDC for which illumina and PacBio sequence data are available in GenBank (BioProject accession # PRJNA218110)*.

b *The stx_2a_stx_2c_ strains contain an stx_1_-like defective phage in yehV*.

c *ND—not determined. Clade type could not be determined with the four SNP typing scheme*.

We used the four gene typing system described by Riordan et al. (2008) and found that the *stx*_2a_+stx_2c_+ and *stx*_2a_+ strains belonged to clade 8. The *stx*_1a_+*stx*_2a_+ strains (JH2014, JH2015) were identified as clade 2, another O157 clade associated with severe human disease (Manning et al., 2008). JH2018 could not be classified with the four gene typing system. With an *E. coli* Multilocus Sequence Typing (MLST) scheme (Alikhan et al., 2018), all nine isolates belong to sequence type 11 and possessed the same virulence gene profiles, including genes related to intimin production (*eae, tir, tccP*), enterohemolysin (*ehxA*), increased serum survival (*iss*), non-LEE effectors (*nleA, nleB, nleC*), Type III secretion proteins (*espA, espB, espF, espD, espJ, espP*), toxin B (*toxB*), and catalase (*katP*). Because the virulence gene profile other than *stx* type was the same among the isolates, we could not correlate any of the non-*stx* genes with disease outcome in the patients.

### The O157:H7 Strains Are Cytotoxic on Vero Cells and Produce Variable Levels of Stx1 and Stx2 *in vitro* After Cip Induction

**Figure 1:**
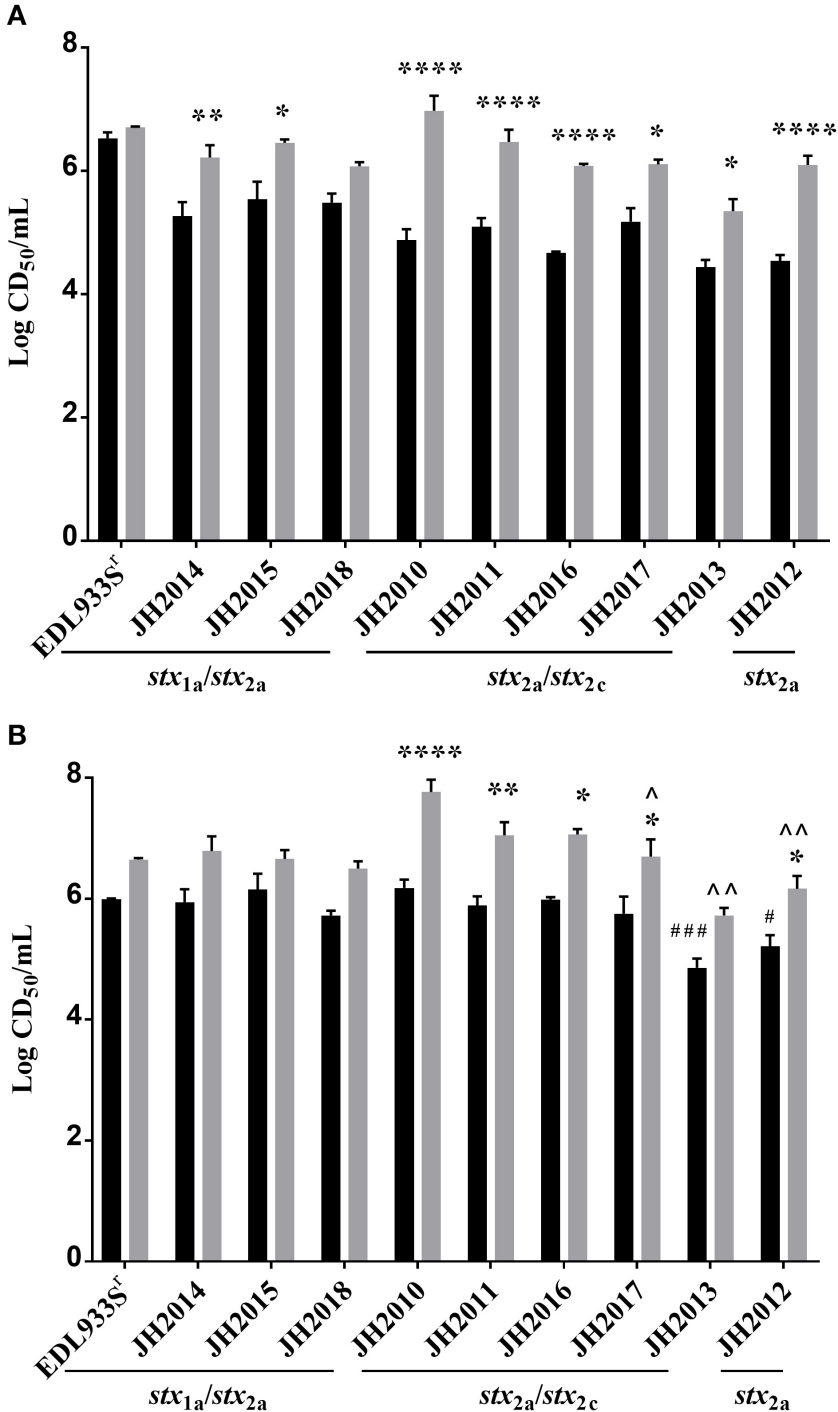
Cytotoxicity of cell-associated or supernatant fractions from cultures of *E. coli* O157:H7 clinical isolates grown in LB with or without Cip. **(A)** Cell-associated and **(B)** supernatant fractions are shown. Results are the mean log CD_50_/ml ± one standard error for at least 5 independent cultures. Two-way ANOVA with Tukey's posttest was used for multiple comparisons. Comparison between no Cip and Cip samples for each individual strain: *P < 0.05; **P < 0.01; ****P < 0.0001. Comparison of cytotoxicity levels in the cell-associated or supernatant no Cip fractions among the strains that make only Stx2: ^#^*P* < 0.05; ^*###*^*P* < 0.001 (as compared to JH2010 no Cip). Cytotoxicity levels in the cell-associated and Cip supernatant fractions compared to JH2010; ^∧^*P* < 0.05. ^∧∧^*P* < 0.0001.

We assessed cytotoxicity of the nine clinical O157:H7 strains and a Str resistant (Str^r^) derivative of EDL933 [a ground beef isolate from the 1983 hemorrhagic colitis outbreak in Michigan (O'Brien et al., 1983)], on Vero cells after the strains were grown in LB or LB supplemented with the *stx*-phage inducer, ciprofloxacin (Cip). The minimum inhibitory concentration for the nine clinical isolates ranged between 20 and 40 ng/ml; therefore, a sub-inhibitory concentration of Cip (5 ng/ml) was used for *stx* induction. After overnight growth in LB with 5 ng/ml Cip there was no statistical difference in CFU count among strains (data not shown). The cell-associated (Figure 1A) or supernatant (Figure 1B) fractions from all the isolates were cytotoxic on Vero cells after growth with or without Cip. The 50% cytotoxic dose (CD_50_) was calculated as the amount of toxin required to kill 50% of the cells in a well. The *stx*_1a_ *stx*_2a_ strains (JH2014, JH2015, and JH2018) exhibited a CD_50_ of ~10^5.5−6^ with and without Cip for both the cell-associated and supernatant fractions. We separated the cell-associated and supernatant fractions, because in some studies Stx1a is more cell-associated and Stx2 is more consistently found in the supernatant (Shimizu et al., 2009). However, since we measured total toxicity, we cannot say how much of each toxin was present in a particular fraction from this assay. We observed a statistical difference in the CD_50_ in the cell-associated fraction between the Cip and no Cip samples for *stx*_1a_*stx*_2a_ strains JH2014 and JH2015 (Figure 1). Cytotoxicity was not induced for strain JH2018, or, alternatively, it is possible that one of the *stx*-phages is inducible, but that measuring the total toxicity does not allow us to detect such an increase if the level of toxin produced from the one phage is higher than the induced levels from the second phage. We confirmed that the *stx*_1a_+ *stx*_2a_+ strains produced both Stx1a and Stx2a by immuno dot blot (data not shown). All strains that produced only Stx2 (we will use Stx2 to mean total Stx2 for strains that may make more than one Stx2 subtype) showed increased cytotoxicity in both the cell-associated and supernatant fractions after growth with Cip, except for JH2013 in the supernatant fraction (Figures 1A,B). Overall, however, we were unable to correlate the *in vitro* cytotoxicity or inducibility of the strains with disease outcome in the patients.

### The Most Virulent O157:H7 Clinical Isolates had the Highest Stx2 Levels in Mouse Stool

**Figure 2:**
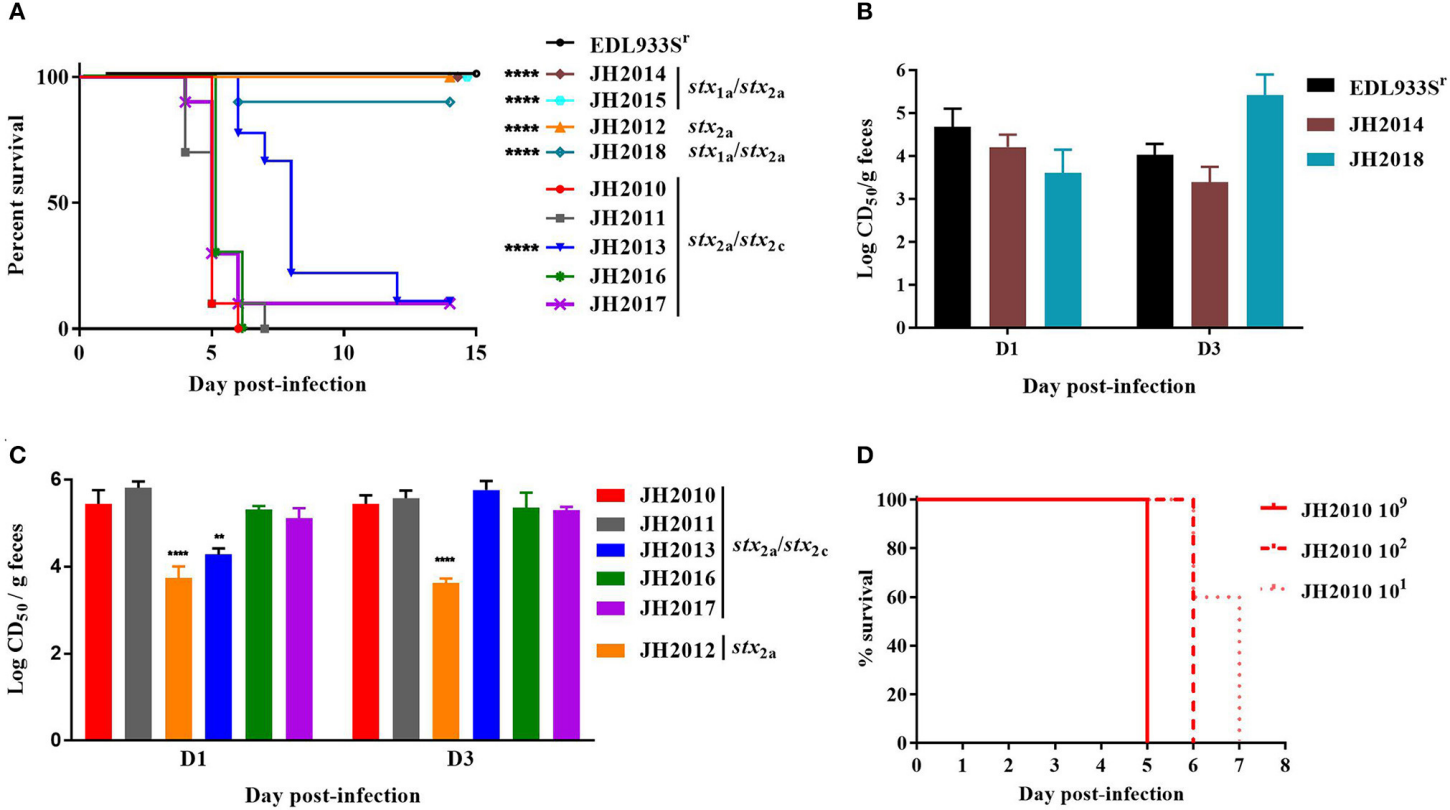
Virulence of clinical O157:H7 strains and cytotoxicity from stool of infected BALB/c mice. **(A)** Survival of Str-treated mice infected with clinical O157:H7 isolates (*n* = 9 or 10 mice per isolate) or control strain EDL933S^r^ (*n* = 10). Survival of mice infected with JH2010 was different than survival after infection with JH2013 and the *stx*_1a_*stx*_2a_ isolates, *P* < 0.0001, indicated by **** on graph. **(B)** Cytotoxicity (mean ± one standard error) of stool supernatant from individual mice (*n* = 5 or 10 mice) infected with either *stx*_1a_*stx*_2a_ strain EDL933S^r^ or JH2014, or JH2018. **(C)** Cytotoxicity from individual mice (*n* = 4 or 5) infected with *stx*_2a_ or *stx*_2a_*stx*_2c_ strains. Error bars represent standard deviation of the mean cytotoxicity. Limit of detection was 10^2^ CD_50_/g feces. **P < 0.01; ****P < 0.0001. **(D)** Survival of Str-treated mice infected with different doses of JH2010 (*n* = 5 mice per dose).

To determine the virulence potential of the clinical strains in the Str-treated mouse model, mice were gavaged with ~10^9−10^ CFU and followed for 14 days. Of the nine clinical strains, the *stx*_2a_+*stx*_2c_+ strains JH2010, JH2011, and JH2016 were lethal in 100% of mice by day 7 post-infection (Figure 2A). Of the mice infected with JH2013 or JH2017 just 10% survived the infection, though there was a significant delay in the death of the mice infected with JH2013 (Figure 2A). The *stx*_1a_s*tx*_2a_ strains JH2014 and JH2015 and the *stx*_2a_-only strain, JH2012 were avirulent. Strain JH2018 (*stx*_1a_s*tx*_2a_) killed 1/9 mice. There were no differences in mouse colonization levels by the strains on day 1 or day 3 post-infection as measured by the number of O157:H7 shed into the feces (data not shown). Although all three of the *stx*_1a_+ *stx*_2a_+ strains were avirulent or slightly virulent, stool from mice infected with these strains exhibited cytotoxicity on Vero cells with a CD_50_ between ~10^3.5^ and 10^5.5^ (Figure 2B). Fecal toxicity data are shown for JH2015 below. Stool from mice infected with the most virulent strains that produced only Stx2 was ~10- to 100-fold more cytotoxic than from avirulent strain JH2012 on days 1 and 3 post-infection and 10-fold more cytotoxic than from the strain with delayed virulence, JH2013, on day 1 post-infection (Figure 2C). We hypothesize from this latter finding that Stx2 from the highly virulent strains is more easily induced in the mouse. Because Stx2 appears to be more easily induced from the virulent strains *in vivo*, we tested lower inoculum doses (10^2^ or 10^1^ CFU) for one strain, JH2010, in the Str-treated mice. We found that although the time-to-death was delayed for the lower doses of bacteria, all of the mice infected with JH2010 succumbed to infection by day 7 post-infection, Figure 2D.

### Differences in Cytotoxicity and Virulence of JH2010 Were Not Due to Differences in Colonization and Inoculum Dose

**Figure 3:**
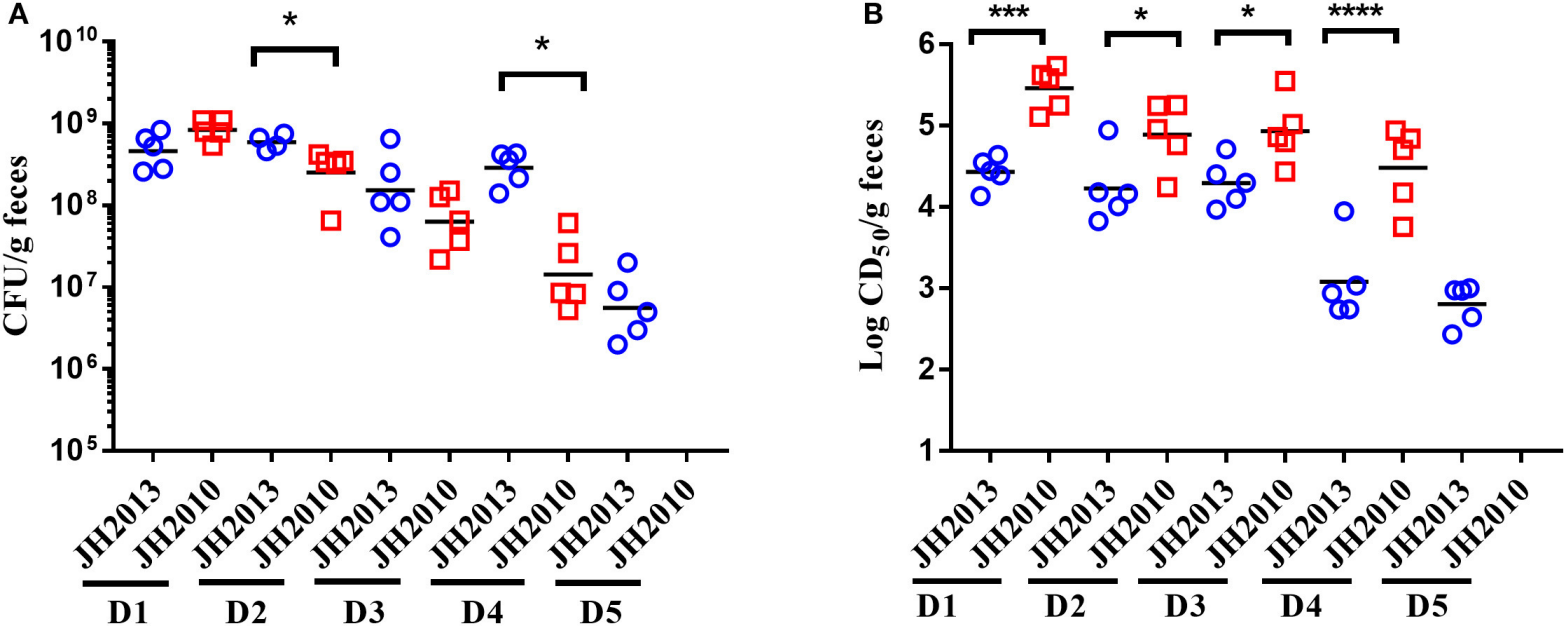
Comparison of colonization and fecal cytotoxicity of two *stx*_2a_+*stx*_2c_+ O157:H7. **(A)** Colonization: stool was collected every day for 5 days (D1–D5) post-infection and plated for colonization (*n* = 5 mice per group) from mice fed 10^9^ CFU JH2013 (circles) or JH2010 (squares). Each symbol represents one animal. The line represents the geometric mean. The limit of detection was 10^2^ CFU/g feces. CFU for each day were compared by unpaired *t*-test. *P < 0.05. **(B)** Cytotoxicity of stool collected from infected mice. Each symbol represents one animal and the line indicates the mean log CD_50_/ml. Cytotoxicity values from the stool samples were compared by unpaired *t*-test. ***P = 0.0005; ****P < 0.0001. Stool could not be collected from mice infected with JH2010 on D5 so colonization and cytotoxicity levels were not determined.

We next did a more extensive comparison of colonization levels over time for JH2010 and the less virulent *stx*_2a_+*stx*_2c_+ strain JH2013. We found that JH2010 colonized similarly or less well than JH2013, as measured by the number of bacteria shed into the feces (Figure 3A). However, the toxicity in stool from mice fed JH2010 was statistically higher on days 1–4 post-infection (Figure 3B). We were unable to compare the toxicity levels in stool on day 5 because too many of the mice had died in the JH2010 group. Taken together, these results suggest that elevated toxin production by JH2010 *in vivo* contributes to the greater virulence of this strain as compared to JH2013.

### *In vivo* Cytotoxicity of Stx1a+ Stx2a+ Strain JH2015 in the Absence of Cip Is Due Mostly to Stx1a

**Figure 4:**
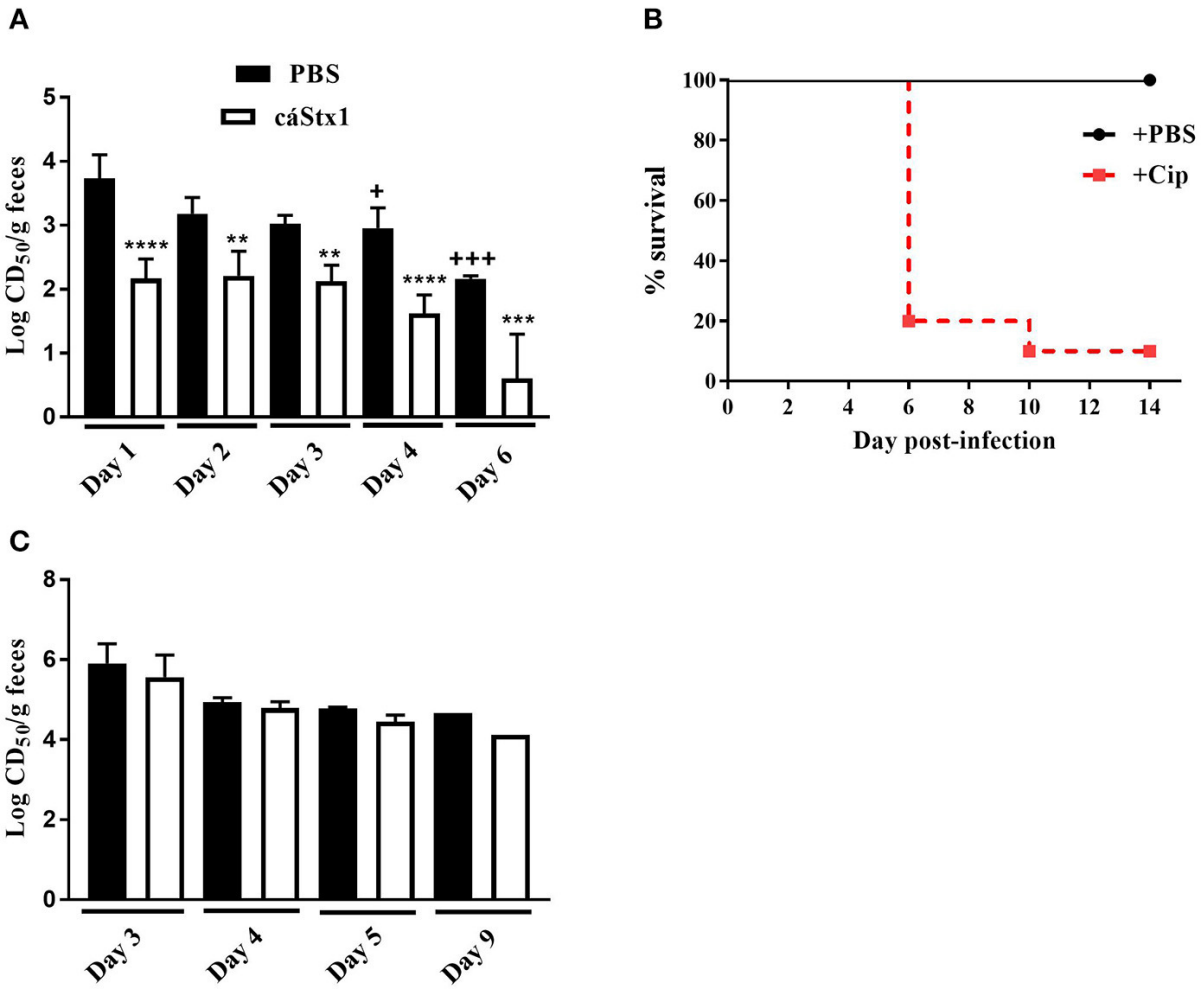
Neutralization of toxin from and survival of Str- or Str- and Cip-treated, JH2015-infected mice. **(A)** Cytotoxicity of stool samples from mice infected with *stx*_1a_*stx*_2a_ strain JH2015 that were incubated with PBS (black bars) or anti-Stx1 antibody, cαStx1 (white bars) (*n* = 5 mice). Results are the mean log CD_50_/g feces ± standard error. One-way ANOVA with Tukey's multiple comparisons test. **P < 0.004; ***P = 0.0006; ****P < 0.0001 (as compared to the sample incubated with PBS). ^+^*P* < 0.05; ^+++^*P* = 0.0002 (compared to the cytotoxicity of stool from day 1 incubated with PBS). **(B)** Survival of Str-treated BALB/c mice infected with JH2015 administered PBS (black line) or 0.1 mL Cip (5 μg/ml; dashed red line) starting on day 2 post-infection. **(C)** Lack of neutralization of Stx1a in stool supernatants from JH2015-infected mice treated parenterally with Cip (5 μg/mouse) beginning on day 2 and administered daily for the remainder of the study. Black bars represent cytotoxicity of fecal supernatants incubated with PBS; white bars represent stool supernatant treated with cαStx1. Results are the mean log CD_50_/g feces ± standard error.

The Stx1a+ Stx2a+ strains JH2015, JH2014, and JH2018 exhibited high levels of colonization (data not shown) and cytotoxicity *in vivo*, similar to that of virulent strain JH2010 however, these strains were avirulent in mice. We hypothesized that the high level of cytotoxicity from stool of mice infected with the avirulent strains was due primarily to Stx1a. To determine the contribution of Stx1a to the cytotoxicity produced by avirulent strain JH2015 *in vivo*, we used an anti-Stx1 antibody to neutralize Stx1a in stool supernatants from feces collected from JH2015-infected mice. Incubation of stool from JH2015-infected mice with anti-Stx1 (100 μg/ml) decreased cytotoxicity of the stool 100-fold, a finding that demonstrated that JH2015 produced primarily Stx1a *in vivo* (Figure 4A). We hypothesize that JH2015 is avirulent in the mice due to the relatively low level of Stx2a produced during infection as indicated by the amount of cytotoxicity that remains after the Stx1a is neutralized (~10^2^ CD_50_/g stool). To determine whether administration of Cip to JH2015-infected, Str-treated mice would increase Stx2 production *in vivo* and induce virulence as was previously observed by Zhang et al. for the Stx2-only producing O157:H7 strain 1:361 (Zhang et al., 2000), we gave Cip (5 μg/mouse) or phosphate-buffered saline (PBS) daily to JH2015-infected mice, beginning on day 2 post-infection until the end of the study. Of the Cip- treated mice, 9 out of 10 succumbed to infection, while all infected mice treated with PBS survived the infection (Figure 4B). Stool supernatants from JH2015-infected Cip-treated mice were 100- to 1,000-fold more cytotoxic on Vero cells than similar samples from mice not treated with Cip (Figure 4C compared to Figure 4A, for example, 10^5.9^ compared to 10^3.0^ CD_50_/g feces on day 3). However, incubation of the stool supernatant from Cip-treated mice with anti-Stx1 did not reduce the toxicity of the samples (Figure 4C); both anti-Stx1 and anti-Stx2 were required for a 1.5-log reduction in toxicity, not shown (The stool samples from the PBS-treated mice in this latter experiment acted similarly to those from untreated mice: the toxicity was neutralized at least 100-fold with anti-Stx1, not shown). These data suggest that treating JH2015-infected mice with Cip increased Stx2a induction and that enhanced level of Stx2 allowed JH2015 to become virulent in mice.

### Deletion of Stx2a in Highly Virulent Strains Attenuates Virulence

**Figure 5:**
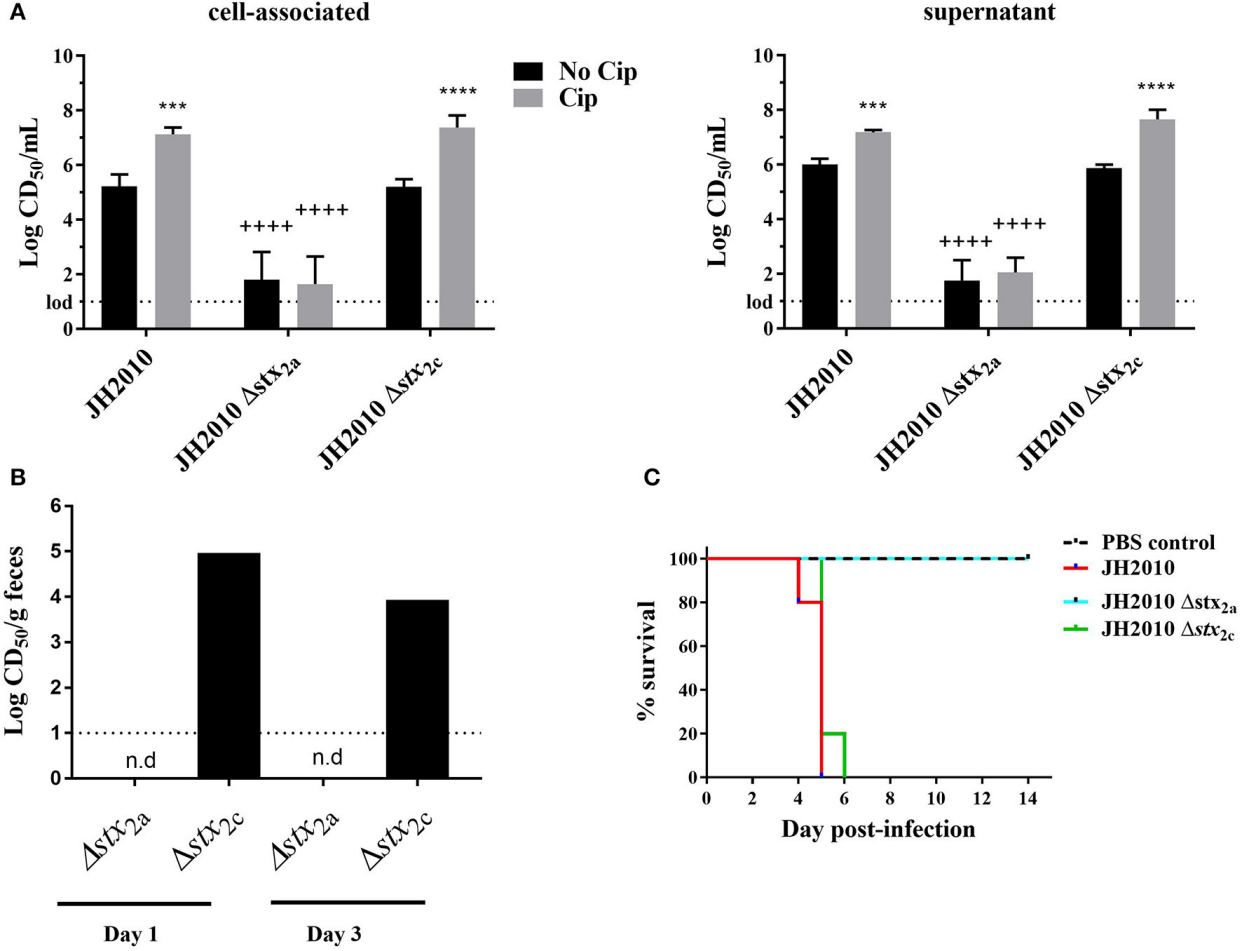
*In vitro* and *in vivo* cytotoxicity and survival of JH2010 *stx*_2a_ and *stx*_2c_ mutants. **(A)** Cytotoxicity produced *in vitro* by the mutants grown in LB or LB with Cip (5 ng/mL). Results are the mean log CD_50_/ml ± standard error for at least five independent cultures. Two-way ANOVA with Tukey's posttest were used for multiple comparisons. ***P < 0.001; ****P < 0.0001 (as compared to the no Cip control). ^++++^*P* < 0.0001 (as compared to the parent strain in the same experimental condition). **(B)** *In vivo* cytotoxicity was determined from pooled stool supernatant from feces collected from each mouse post-infection. No cytotoxicity was detected in *stx*_2a_ mutants (n.d). Bar represents cytotoxicity of pooled feces from five mice. **(C)** Virulence of *stx*_2_ mutants in Str-treated BALB/c mice. JH2010 (*n* = 15 mice), mutant strains (*n* = 5 mice per strain).

JH2010 is lysogenized by an *stx*_2a_ and an *stx*_2c_ phage. To determine whether one or both of the *stx*s plays a role in the virulence of the strain, we generated *stx*_2a_ and *stx*_2c_ deletion mutants in the JH2010 background. Supernatant and cell-associated fractions from the *stx*_2c_ mutant grown with or without Cip were as cytotoxic *in vitro* as the parental strain (Figure 5A). In contrast, deletion of *stx*_2a_ caused a >1,000-fold decrease in cytotoxicity when the mutant was grown with or without Cip (Figure 5A). These latter results suggest that a low level of Stx2c is produced from *stx*_2c_ but that expression is not induced with Cip *in vitro* (Figure 5A). *In vivo*, stool supernatant from feces of JH2010Δ*stx*_2c_-infected mice displayed similar levels of cytotoxicity as the parental strain on Vero cells (Figure 5B) and was equally virulent in mice (Figure 5C). In contrast, stool supernatants from feces of JH2010Δ*stx*_2a_-infected mice were not cytotoxic (Figure 5B) and the *stx*_2a_ mutant was avirulent in mice (Figure 5C). These data indicate that Stx2a is responsible for the virulence of JH2010 in Str-treated mice. We observed similar results for another *stx*_2a_+*stx*_2c_+ strain, JH2011 (Supplemental Figure 1). Taken together our results show that Stx2a is responsible for the virulence of both JH2010 and JH2011.

### RecA-Dependent Induction of *stx*_2_ Phage Is Required for Virulence of JH2010 *in vivo*

**Figure 6:**
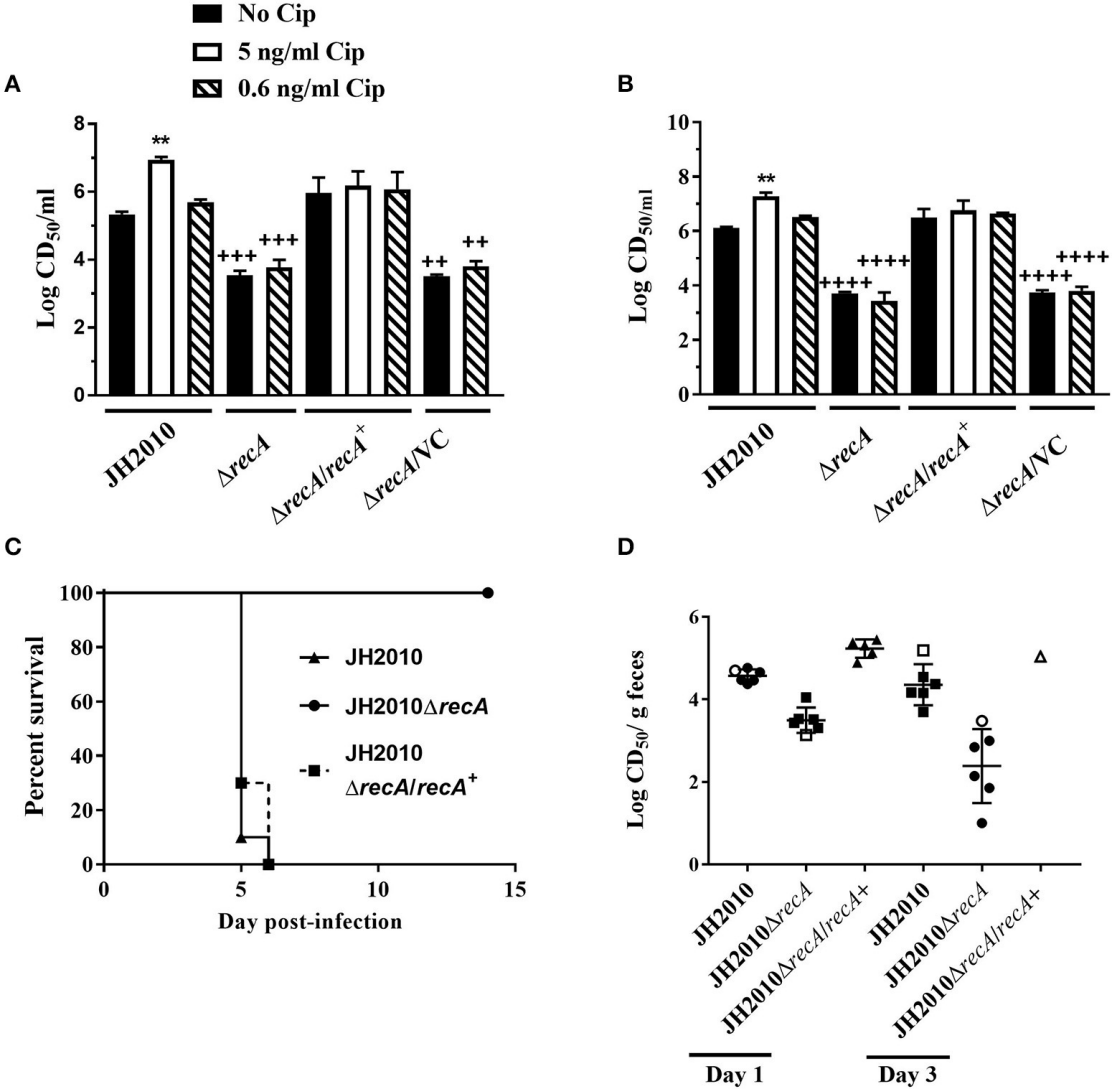
RecA-dependent induction of Stx in JH2010. Cytotoxicity of JH2010, JH2010Δ*recA*, JH2010Δ*recA/recA*^+^, and JH2010Δ*recA/*VC *in vitro* of cell-associated **(A)** and the supernatant **(B)** fractions. Results are the mean log CD_50_/ml ±standard error for at least five independent cultures. One-way ANOVA with Tukey's posttest were used for multiple comparisons. **P < 0.01 (as compared to the no Cip control). ^++^*P* < 0.01; ^+++^*P* < 0.001; ^++++^*P* < 0.0001 (as compared to the parent strain in the same experimental condition). **(C)** Survival of mice infected with JH2010, JH2010Δ*recA*, or JH2010Δ*recA/recA*+. **(D)** Cytotoxicity of stool supernatant from feces of mice infected with JH2010, the *recA* mutant, or the complemented strain. Each solid symbol represents one animal, open symbols represent pooled samples from a cage of five mice. The line is the mean log CD_50_/g feces from the stool supernatant. One-way ANOVA with Tukey's posttest were used for multiple comparisons. *P < 0.05; **P < 0.01; ***P < 0.001; ****P < 0.0001 (as compared to the no Cip control).

To determine whether virulence of JH2010 is due to RecA-dependent induction of the s*tx*_2_ phages, we generated a *recA* deletion mutant. On agar plates, colonies of the *recA* deletion mutant appeared smaller compared to the parental strain; however, the mutant did not exhibit a growth defect when grown in LB broth (data not shown). The *recA* mutant also displayed increased sensitivity to Cip with a minimum inhibitory concentration (MIC) of 2.5 ng/ml compared to the parent (40 ng/ml). Complementation of *recA* expressed from its native promoter restored both colony morphology and the MIC for Cip. *In vitro*, deletion of *recA* reduced cytotoxicity on Vero cells of both the cell-associated and supernatant fractions ~100- fold when grown in LB or LB-Cip (0.6 ng/ml; Figures 6A,B). The *recA* mutant strain colonized Str-treated mice to similar levels as the parent strain (data not shown); however, the mutant was avirulent (Figure 6C). Str-treated mice fed the complemented mutant strain succumbed to infection by day 5 (Figure 6C). Cytotoxicity of stools collected from mice infected with the Δ*recA* mutant were 10- to 100-fold lower compared to the parent (Figure 6D). These data suggest RecA-dependent induction of *stx*_2_ phage in JH2010 occurs *in vivo* and is necessary for the virulence of the strain.

## Discussion

In this study, we found that four STEC strains from a collection of nine O157:H7 isolates were highly virulent in Str-treated mice. One of those strains, JH2010, killed 100% of Str-treated infected mice even at an inoculum of 10^1^ CFU, and lethality was dependent on Stx2 and RecA. Although JH2010 possesses the genes for both *stx*_2a_ and *stx*_2c_, toxicity could not be detected in stool from mice infected with JH2010Δ*stx*_2a_, and that mutant was avirulent. Taken together these findings indicate that Stx2a is entirely responsible for the high virulence of JH2010. The toxicity of JH2010 and the other highly virulent strains identified in this study was 10- to 100-fold higher in the feces from infected mice as compared to avirulent strain JH2012. We hypothesize that the higher levels of toxin in the stool from mice infected with the highly virulent O157:H7 isolates is an indication that the *stx*_2a_-phage from those strains is readily induced *in vivo*. A study to compare the induction of *stx*_2a_-phage in the stool from mice infected with JH2010 or JH2012 would allow us to address this latter hypothesis.

Our finding that the virulence of JH2010 is dependent on RecA is consistent with a previous study that showed RecA is required for the virulence of STEC isolates EDL933 (Stx1a+ Stx2a+) and 86-24 (Stx2a+) in an intravenous infection model and lung toxicity assay (Fuchs et al., 1999). Furthermore, the *stx*_2a_-phage from EDL933 is induced in germ-free mice, a finding that also suggests a requirement for RecA (Tyler et al., 2013) even in the absence of antibiotic treatment to induce the phage. Our findings further indicate that some STEC exhibit enhanced induction of *stx*_2a_-encoding bacteriophage in the mouse intestine. Production of Stx2a in strains JH2010 and JH2011 (not shown) was predominately RecA-dependent. Deletion of *recA* rendered both strains avirulent and decreased cytotoxicity recovered from stool of infected mice. In these strains, there is RecA-independent expression and release into the supernatant of Stx2 *in vitro* and *in vivo*. These latter findings demonstrate that there is a non-RecA-mediated way to produce and release Stx2a, a result that contrasts to findings for O157:H7 strain 86-24 in which there was only a low level of Stx2 in the cell-associated fraction in the 86-24 RecA mutant and no Stx2 in the supernatant after growth in LB (Fuchs et al., 1999). However, the level of Stx2a produced in the absence of RecA is not sufficient for lethality in Str-treated mice. We hypothesize that the RecA-independent expression may be due to increased sensitivity to oxidative stress or other factors that lead to expression of Stx2 or induction of the *stx*_2_-phage in a RecA-independent manner, as found after growth of cultures in the presence of ethylenediaminetetraacetate (EDTA) (Imamovic and Muniesa, 2012).

Although Stx2a levels were induced from JH2012 after growth with Cip *in vitro*, and Stx2a was detected in the stool of Str-treated, JH2012-infected mice, JH2012 was avirulent. While it is possible that JH2012 produces a factor that reduces virulence in Str-treated mice, we deem it more likely that mutations exist within the *stx*_2a_-phage or the host bacterial genome of JH2012 that prevent complete cell lysis or toxin release from the cell that would be needed for this strain to exhibit mouse pathogenicity. Indeed, another laboratory demonstrated that both phage and bacterial sequences influence toxin production and release *in vitro* (Yin et al., 2015). Moreover, differences in Stx2 production even among clade 8 strains has previously been reported (Neupane et al., 2011). We compared the sequence upstream of *st*x_2a_ from JH2012 with those of JH2011, JH2016, and JH2017 (not shown) and found the same SNPs in the *q* region upstream of *stx*_2a_ in JH2012 that have been identified in clade 8 strain EC508 as described by Neupane et al. (2011). However, because the same *q* region SNPs were found in other clade 8 STEC, and those strains express different levels of Stx2 *in vitro* (Neupane et al., 2011), the *q* region SNPs alone cannot explain the differences in Stx2a levels we observed *in vivo* for JH2012 as compared to the *stx*_2a_*stx*_2c_ O157:H7 clade 8 strains in this study. However, despite the differences in induction after growth with cip between strain JH2012 and the more virulent strains, we hypothesize that Cip-treatment of mice infected with JH2012 would result in death of the animals because the levels of Stx2a produced *in vitro* by JH2012 after induction are similar to those from JH2013. The *q* regions for the virulent strains JH2011, JH2016, and JH1017 are identical to those of the majority of clade 8 strains as described (Neupane et al., 2011).

We observed that the clinical Stx1a+ Stx2a+ strains were avirulent in mice. Neutralization of Stx1a from stool homogenates of mice infected with those Stx1a+ Stx2a+ strains revealed that Stx1a is the predominant toxin produced by these strains *in vivo*. The relatively low levels of Stx2a made by these strains *in vivo* was surprising to us but could be related to the insertion site for the *stx*_2a_-phage in these strains (*wrbA*) or possibly to the presence of the *stx*_1a_-phage. It has been demonstrated that the presence of two *stx*_2_ phages within a single bacterial K12 host lowers the level of toxin produced compared to singly lysogenized strains (Serra-Moreno et al., 2008). Nevertheless, mice infected with Stx1a+ Stx2a+ strain JH2015 and then treated with Cip released high levels of Stx2a into their stool and succumbed to the infection. We attribute the enhanced virulence of the Stx1a+ Stx2a+ strain JH2015 in Cip-treated infected mice to increased production of Stx2a because neutralization of the toxicity in the stool from infected and Cip-treated mice required both Stx1 and Stx2 antibody, a fact that indicates that the Cip treatment greatly enhanced the Stx2a levels *in vivo* as compared to PBS-treated mice, a phenomenon previously observed in an Stx2-only O157:H7 strain (Zhang et al., 2000). We do not know whether the lack of virulence of the Stx1a+Stx2a+ isolates in the mouse model is due to the relatively low level of Stx2a production in the absence of Cip treatment or if the presence of Stx1a is protective in these strains. Our laboratory previously demonstrated in co-intoxication studies that Stx1a reduces Stx2a mediated toxicity *in vivo* particularly when more Stx1a than Stx2a is administered (Russo et al., 2016). We also recently found that the presence of Stx1a reduces the morbidity in Str-treated mice after infection with an STEC strain that makes both Stx1a and Stx2a (Petro et al., 2019).

In our subset of strains, we found that Stx2c did not contribute to virulence, because deletion of *stx*_2a_ in all of the *stx*_2a_ *stx*_2c_ isolates resulted in at least a 1,000-fold reduction of toxicity *in vitro* and complete attenuation in mice. The finding that Stx2c is produced at low levels in some isolates coincide with previous observations (Strauch et al., 2004; Ogura et al., 2015). However, there are other strains that do produce enough Stx2c to be moderately pathogenic in Str-treated mice (Lindgren et al., 1994). We were able to induce Stx2c *in vivo* in the *stx*_2a_ phage-cured strain of JH2010 after Cip-treatment of the mice (data not shown). All the strains lysogenized by an *stx*_2c_-phage shared 100% sequence identity to *stx*_2c_ bacteriophage 2851, which is the phage considered to be the progenitor phage for this subtype (Strauch et al., 2004). We also note that *stx*_2a_ in JH2012 occupies the *argW* site, a site usually occupied by *stx*_2a_ in strains that are also lysogenized by an *stx*_2c_-phage in the *sbcB* site. One possibility for this unexpected finding is that JH2012 lost an *stx*_2c_ phage.

In this study we initially evaluated factors, such as *stx* subtypes, *stx*-phage insertion sites, and virulence in Str-treated mice for nine clinical isolates to determine whether these factors could be correlated with severity of human disease. We were unable to make such a correlation, perhaps due to the relatively low number of strains in our study. It might be possible to correlate the *stx* subtypes and *stx*-phage insertion sites of O157:H7 strains to the clinical disease manifestation if a large collection of complete whole genome sequences became available for isolates with corresponding clinical data.

However, we did find that the level of increase in cytotoxicity from the strains in response to growth in Cip, or after infection in Str-treated mice varied among the isolates. Furthermore, we demonstrated that the best predictor of virulence in the mice was high levels of Stx2a in the stool. In contrast, one study in humans found that Stx in stool at day 4 or later illness manifestation was inversely correlated with disease (Cornick et al., 2002); however, those authors also suggested that toxin levels were likely high early in infection and then was absorbed. We found that lower levels of Stx2a in stool led to delayed virulence or avirulence in mice. It may be that at lower levels of Stx2a, host factors or other bacterial factors play a role in virulence, and such variables likely also have roles in human disease. However, our results demonstrate that the level of *in vivo* of induction of the *stx*_2_-phage contributes to the severity of disease in mice.

## Materials and Methods

### Bacterial Strains, Plasmids, and Culture Growth Conditions

All strains and plasmids used in this study are described in Table 2. *E. coli* O157:H7 strains isolated from patients who developed HUS (*n* = 3), bloody diarrhea (*n* = 3) or non-bloody diarrhea (*n* = 3) came from the CDC *Escherichia* and *Shigella* Reference Unit (Centers for Disease Control and Prevention, Atlanta, Ga) and were demonstrated by that group to be agglutinated by both anti-O157 and anti-H7 serum. Isolates were grown at 37°C on Luria-Bertani (LB) agar, Sorbitol-MacConkey (SMAC) agar, or in LB broth with aeration. Spontaneous Str-resistant (Str^r^) strains were isolated and used for all *in vitro* and animal studies. For antibiotics, unless stated otherwise, the following concentrations were used: ampicillin (Ap; 100 μg/ml); chloramphenicol (Cm; 30 μg/ml), ciprofloxacin (Cip; 5 ng/ml), Str (50 μg/ml), and tetracycline (Tc; 10 μg/ml).

**Table 2:** Bacterial strains and plasmids used in this study.

**Strain**	**Description**	**References/Source**
***E. coli*** **strains**		
EDL933S^r^	*E. coli* O157:H7 ${stx}_{1\text{a}}^{+}$ ${stx}_{2\text{a}}^{+}$ strain; Str^r^ derivative of EDL933	O'Brien et al., 1983, This study
JH2010	*E. coli* O157:H7 clinical strain; Str^r^ derivative of CDC# 06-3462	This study
JH2011	*E. coli* O157:H7 clinical strain; Str^r^ derivative of CDC# 08-3914	This study
JH2012	*E. coli* O157:H7 clinical strain; Str^r^ derivative of CDC# 08-3918	This study
JH2013	*E. coli* O157:H7 clinical strain; Str^r^ derivative of CDC# 2009C-3378	This study
JH2014	*E. coli* O157:H7 clinical strain; Str^r^ derivative of CDC# 2009C-3554	This study
JH2015	*E. coli* O157:H7 clinical strain; Str^r^ derivative of CDC# 2009C-4207	This study
JH2016	*E. coli* O157:H7 clinical strain; Str^r^ derivative of CDC# 2009C-4687	This study
JH2017	*E. coli* O157:H7 clinical strain; Str^r^ derivative of CDC# 2010C-3142	This study
JH2018	*E. coli* O157:H7 clinical strain; Str^r^ derivative of CDC# 2010C-3347	This study
JH2026	JH2011 Δ*stx_2*a*_*, Str^r^, Cm^r^	This study
JH2028	JH2011 Δ*stx_2*c*_*, Str^r^, Cm^r^	This study
JH2030	JH2010 Δ*stx*_2c_, Str^r^, Cm^r^	This study
JH2031	JH2010 Δ*stx*_2a_, Str^r^, Cm^r^	This study
JH2047	JH2010 Δ*recA*, Str^r^, Cm^r^	This study
JH2058	JH2010 Δ*recA* (JH2047) complemented with pJH206; referred to as Δ*recA*/*recA*^+^ in text; Str^r^ Cm^r^, Ap^r^	This study
JH2059	JH2010 Δ*recA* (JH2047) complemented with pACYC177 referred to as Δ*recA*/VC in text; Str^r^ Cm^r^, Ap^r^	This study
TOP10	F^−^*mcr*A Δ(*mrr*-*hsd*RMS-*mcr*BC) Φ80 *lac*ZΔM15 Δ*lac*X74 *rec*A1 *ara*D139 Δ(*ara*-*leu*)7697 *gal*U *gal*K *rps*L (Str^R^) *end*A1 *nup*G	Invitrogen
**Plasmids**		
pACYC177	Low copy number plasmid; Km^r^, Ap^r^	Invitrogen
pCR™2.1-TOPO^®^	PCR cloning vector; Km^r^, Ap^r^	Invitrogen
pHSG3962	pUC vector, Cm^r^	Takara Bio USA, Inc.
pJH203	*recA* + 500bp upstream pCR II-TOPO vector; Cm^r^, Km^r^, Ap^r^	This study
pJH206	*recA* + 500bp upstream fragment from pJH203 digested with *Xho*I/*Hin*dIII and ligated to pACYC177; Cm^r^, Km^s^, Ap^r^	This study

### stx Subtypes, Phage Insertions Sites, and Clade Determination

DNA was extracted with the Wizard® Genomic DNA Purification kit (Promega) according to manufacturer's protocol. PCR was used to determine the *stx* subtypes (Scheutz et al., 2012) and *stx*-phage chromosomal insertion sites (Scheutz et al., 2012; Bonanno et al., 2015) as described in those references. MG1655 was used as the negative control strain used for the *stx*-phage insertion sites. The positive control strains used for *stx*-phage insertion site determination were as follows: EDL933 (*wrbA, yehV*), EC4045 (*sbcB, argW*), and B2F1 (*yecE*). The phage insertion sites were confirmed by analysis of the whole genome sequence. Strains were assigned to clades based on known polymorphic SNPs previously described (Riordan et al., 2008).

### Sequencing

Pacific BioSciences sequencing was completed as previously described (Lindsey et al., 2017). Briefly, *E. coli* genomic DNA was extracted according to the manufacturer's protocol (Archive Pure, 5 Prime, Gaithersburg, MD). The DNA was sheared to 20 kb fragments using needle shearing and Blue Pippin was used for size selection. DNA fragments were used to generate large SMRTbell™ libraries using the standard library protocols of the Pacific Biosciences DNA Template Preparation Kit (Menlo Park, CA). One SMRTcell was used to sequence each isolate. Finished libraries were bound to proprietary P6v2 polymerase and sequenced on a PacBio RSII sequencer using C4 chemistry for 360 min movies. Sequence reads were filtered and assembled *de novo* with the PacBio Hierarchical Genome Assembly Process version 3 and polished using Quiver (Chin et al., 2013). The same DNA extract for all isolates except for 08-3914 and 2009C-4687 were sequenced with an illumina MiSeq following manufacturers protocols (Illumina, USA). The PacBio sequences (except for 08-3914 and 2009C-4687) were Illumina corrected with unicycler_polish that uses Pilon (Walker et al., 2014; Wick et al., 2017).

Genomic analysis was done with CLC Genomics Workbench 9.5.3 (https://www.qiagenbioinformatics.com). Even though whole genome sequence was generated for each of the isolates, for strains 06-3462 (progenitor to JH2010) and 2009C-3378 (progenitor to JH2013), the *stx*_2_-phage sequences were not fully resolved. Virulence gene profiles of clinical strains were determined using Virulence finder (https://cge.cbs.dtu.dk/services/VirulenceFinder). MLST typing was assigned by uploading sequences for seven housekeeping genes (*adk, fumC, gyrB, icd, mdh, purA*, and *recA*) to The Enterobase MLST database at the University of Warwick [https://enterobase.warwick.ac.uk/species/ecoli/allele_st_search (Alikhan et al., 2018)].

### Stx Mutations

#### Transformation of O157 Strains

The λ Red-mediated gene replacement protocol previously described was used for mutagenesis of O157:H7 strains (Murphy and Campellone, 2003). The Red + Gam-producing plasmid, pTP223, which confers Tc resistance, was electroporated into each isolate (2.5 V; 200 Ω) and plated on LB-Tc.

*stx*_2_ mutagenesis: For deletion of *stx*_2_, *cat*, which confers resistance to Cm, was amplified by PCR from pHSG3962 (linearized using *Hin*dIII/*Bam*HI) with primers Stx2aCMF (5′- ATGAAGTGTATATTATTTAAATGGGTACTGTGCCTGTTACTGGGTTTTTCGCACGTAAGAGGTTCCAACTTTCACCATAATG-3′) and Stx2aCMR (5′-TTCAGCAAATCCGGAGCCTGATTCACAGGTACTGGATTTGATTGTGACAGtTACGCCCCGCCCTGCCACTCATC-3′) that contain 5′ and 3′ overhangs homologous to the first and last 50 base pairs of *stx*_2_. Strains with pTP223 that were grown overnight in LB-Tc were diluted 1/50 in LB-Tc with 10 mM IPTG and grown to OD_600_ ~0.2. The culture was placed on ice for 10 min, then washed and resuspended in 3-(N-Morpholino) propanesulfonic acid (MOPS) with 20% glycerol and made electrocompetent as mentioned above. Five or 10 μl of PCR product were electroporated into competent cells and incubated at 37°C with aeration in Super Optimal broth with Catabolite repression (S.O.C) media (Invitrogen) for 2 h or overnight. Transformants were selected for Cm^r^ and screened for Tc-sensitivity to ensure that the recombineering plasmid was lost. PCR and southern blot were used to confirm deletion of *stx*_2a_ or *stx*_2c_.

*recA* mutagenesis: *recA* in O157:H7 strain JH2010 was replaced with *cat*. *cat* with 5′ and 3′ overhangs homologous to the first and last 50 base pairs of *recA* was amplified by PCR from pHSG3962 with primers recAF3 (5′-TTAAAAATCTTCGTTAGTTTCTGCTACGCCTTCGCTATCATCTACAGAGAAATTACGCCCCGCCCTGCCACTCATC-3′) and recAR4 (5′-atgGCTATCGACGAAAACAAACAGAAAGCGTTGGCGGCAGCACTGGGCCAGCACGTAAGAGGTTCCAACTTTCACCATAATG-3′). The PCR product was electroporated into competent cells and Cm^r^ transformants were selected and screened for Tc-sensitivity to ensure that the recombineering plasmid was lost. Two independent mutants were generated. Mutants were confirmed by PCR and Southern blot (not shown). *recA* mutants were complemented with *recA* expressed from its native promoter cloned into the low copy plasmid, pACYC177. The construct was generated by PCR amplification of *recA* including 500 bp upstream of the *recA* start site, which includes a hypothetical protein with primers recA_upF (5′-ATGGCTATCGACGAAAACAAAC-3′) and recAR5 (5′-GTATCAAACAAGACGATTAAAAATCTTCG-3′). The PCR fragment was cloned into pCR™2.1-TOPO® vector, digested using *Hin*dIII and *Xba*I, and then ligated to pACYC177. The resulting plasmid, pJH206, was transformed into the JH2010 Δ*recA* mutant, and Ap^R^ colonies were selected. As a control, parent strains were transformed with an empty pACYC177 vector (denoted as Δ*recA*/VC in the figure legends). For all assays, complement and vector control (VC) strains were grown in Ap-containing media.

#### Vero Cell Assay

Overnight cultures were diluted 1/500 into LB or LB ± Cip (5 ng/ml) and grown overnight. Cells were pelleted by centrifugation at 15,871 × g for 5 min. The supernatants were filter-sterilized with a 0.22 μm filter. The cell pellets were resuspended in an equal volume of water and subjected to three freeze-thaw cycles. The cell-associated fraction and supernatants were serial-diluted in Eagle's Minimal Essential Medium and 100 μl of each dilution was overlaid onto Vero cells that were seeded in microtiter plates 24 h previously. The Vero cell plates were incubated for 48 h at 37°C in 5% CO_2_, then the media was removed and the cells were then fixed in 10% formalin solution and stained with 0.13% crystal violet. The optical density was measured at 630 nm on BioTek (Winooski, VT, USA) EL800 spectrophotometric plate reader. The 50% cytotoxic dose (CD_50_) represented the amount of toxin required to kill 50% of the cells in a well.

#### Mouse Studies

All mouse studies were conducted in accordance with the recommendations of the Guide for the Care and Use of Laboratory Animals and were approved by the Institutional Animal Care and Use Committee of the Uniformed Services University. Male BALB/c mice (weight, 14 g) from Charles River Labs (Wilmington, MA) were used for all mouse experiments. Mice were fed drinking water containing Str (5 g/L) and fasted for ~18 h prior to infection. Mice were infected by oral gavage with 100 μl of ~10^10−11^ CFU bacteria or PBS. Food was returned after infection and Str water was continued for the remainder of the experiment.

Stool samples were suspended in PBS 1:10 (weight/volume) and homogenized. To determine colonization, supernatants from homogenized stool were serially diluted, plated on SMAC-Str, and incubated overnight at 37°C. Colonies were enumerated to determine the number of CFU/g feces. The limit of detection was 10^2^ CFU. To determine cytotoxicity *in vivo*, stool homogenates were centrifuged for 10 min at 15,871 × g, the supernatants were collected, serially diluted and a Vero cell assay was done as described above. Mice were monitored for morbidity and mortality for 14 days post-infection. Infected mice were euthanized if they displayed two of the following morbidity symptoms: 25% weight loss, lethargy, ruffled fur, difficulty breathing, and/or difficulty moving.

#### Neutralization of Stx1 in Stool Samples

To determine the amount of Stx1a present in fecal samples, stool supernatants (as described above) from infected mice were diluted 1/5 in EMEM then mixed 1:1 with EMEM containing cαStx1 (100 μg/ml), a mouse-human chimeric monoclonal antibody against Stx1 (Edwards et al., 1998), then incubated for at 37°C in 5% CO_2_ for 2 h. Finally, 100 μl of toxin-antibody mixture was overlaid onto Vero cells and the cytotoxicity assay was completed as above.

#### Statistical Analyses

All statistical analyses were done with GraphPad Prism v7.03 for Windows software (GraphPad Software, San Diego, CA).

#### Accession Number(s)

The sequences for the nine clinical isolates used in this study are included in BioProject accession # PRJNA218110.

## Data Availability Statement

The whole genome sequences for the nine O157:H7 isolates from the CDC described in this study are available through GenBank as part of BioProject PRJNA218110.

## Ethics Statement

All mouse studies were conducted in accordance with the recommendations of the Guide for the Care and Use of Laboratory Animals and were approved by the Institutional Animal Care and Use Committee of the Uniformed Services University.

## Author Contributions

JH, NS, AO'B, and AM-C conceived this study. JH, CP, and RA conducted the experiments. RL and NS were responsible for sequencing. JH and AM-C wrote the manuscript with the support of all authors.

### Conflict of Interest

The authors declare that the research was conducted in the absence of any commercial or financial relationships that could be construed as a potential conflict of interest.
