# Supplementary material for: The Virulence of Escherichia coli O157:H7 Isolates in Mice Depends on Shiga Toxin Type 2a (Stx2a)-Induction and High Levels of Stx2a in Stool
Source: Front Cell Infect Microbiol. 2020 Feb 26;10:62. doi: 10.3389/fcimb.2020.00062 (PMC7054288; doi:10.3389/fcimb.2020.00062)
Supplement: Supplementary file 1 [file Data_Sheet_1.docx]

**Supplemental Fig. 1. Deletion of *stx*_2a_ or *stx*_2c_ in virulent strain JH2011.** (A) Cytotoxicity of the cell-associated and supernatant fractions from the mutants grown in LB or LB with Cip (5 ng/mL). Results are the mean Log CD_50_/ml ± standard error for at least 5 independent cultures. Two-way ANOVA with Tukey’s posttest was used for multiple comparisons. ****, *P* <0.0001 (as compared to the no Cip control for the same strain). ++++, *P* <0.0001 (as compared to the parent strain in the same experimental condition). (B) Virulence of JH2011 toxin mutants in Str-treated BALB/c mice (n=5 mice per strain). The survival curves for JH2011 Δ*stx*_2c_ was statistically different than the survival curve for JH2011 Δ*stx*_2a_, *P* = 0.005, with the Log-rank (Mantel-Cox test). (C) Cytotoxicity of stool from infected mice. No cytotoxicity was detected (n.d) in feces collected from mice infected with the *stx*_2a_ mutant. The bar represents the total cytotoxicity of pooled feces from five mice. Dotted line represents the limit of detection (lod) for the assay.
